# Primary treatment patterns in women recruited to the Australian Ovarian Cancer Study

**DOI:** 10.1186/1897-4287-10-S2-A80

**Published:** 2012-04-12

**Authors:** J Hung, S Fereday, P Harnett, D Giles, B Gao, N Traficante, G Chenevix-Trench, A Green, P Webb, D Bowtell, A DeFazio

**Affiliations:** 1 Department of Gynaecological Oncology, University of Sydney at Westmead Millennium Institute, Westmead Hospital, Westmead, NSW, Australia; 2 Department of Medical Oncology and Palliative Care, University of Sydney at Westmead Millennium Institute, Westmead Hospital, Westmead, NSW, Australia; 3 Westmead Institute for Cancer Research, University of Sydney at Westmead Millennium Institute, Westmead Hospital, Westmead, NSW, Australia; 4 Peter MacCallum Cancer Centre, East Melbourne, VIC, Australia; 5 Queensland Institute of Medical Research, Brisbane, Qld, Australia; 6 Department of Biochemistry and Molecular Biology, University of Melbourne, Parkville, VIC, Australia

## 

Associations between clinical outcome and patient characteristics, such as tumour gene expression, and inherited variation (single nucleotide polymorphisms) can be difficult to reproduce between ovarian cancer cohorts. Sources of variation include the size and composition of patient cohorts and treatment, which has varied over time and can vary between countries. We have undertaken a review of treatment in patients recruited to the Australian Ovarian Cancer Study (AOCS) to determine the primary treatment patterns and levels of consistency across the cohort. Australian clinical practice guidelines for the management of women with epithelial ovarian cancer were published in 2004 [1] and AOCS recruited women with suspected ovarian, fallopian tube or primary peritoneal cancer from 2002-2006.

AOCS collected fresh tissue, blood, epidemiological/dietary questionnaires and clinical follow-up data for women aged 18-79 diagnosed with invasive (n=1476) and borderline (n=352) cancer, and material and data are available for research, by application. Clinical data was collected through a national research nurse network at pre-specified intervals: at diagnosis, completion of primary treatment and then at 6-monthly intervals. At the end of Feb 2011 primary treatment data was complete on 99% of eligible cases and the median follow-up time was 5.1 years.

Most patients with invasive cancer underwent surgery prior to chemotherapy (n=1190, 82%) and a small proportion had an interval debulk (surgery following ~3 cycles of chemotherapy, 12%) or other procedure (<10%). The vast majority of women with advanced stage cancer (FIGO stage II-IV) received chemotherapy (1116/1167, 96%) and of these, over 99% (1110/1116) received a platin-based regimen. The most common regimens were carboplatin/paclitaxel (77%) and carboplatin alone (9%), most women (86%) received 6-8 cycles and 95% of women who received adjuvant chemotherapy began treatment within 6 weeks of surgery.

In conclusion, the majority of women in AOCS were treated according to national guidelines, enabling the selection of cases with uniform treatment for projects investigating associations of genomic and genetic features with clinical outcome.
